# The Effects of High-Intensity versus Low-Intensity Resistance Training on Leg Extensor Power and Recovery of Knee Function after ACL-Reconstruction

**DOI:** 10.1155/2014/278512

**Published:** 2014-04-27

**Authors:** Theresa Bieler, Nanna Aue Sobol, Lars L. Andersen, Peter Kiel, Peter Løfholm, Per Aagaard, S. Peter Magnusson, Michael R. Krogsgaard, Nina Beyer

**Affiliations:** ^1^Musculoskeletal Rehabilitation Research Unit, Bispebjerg and Frederiksberg Hospitals, University of Copenhagen, Bispebjerg Bakke 23, 2400 NV Copenhagen, Denmark; ^2^Institute of Sports Medicine, Copenhagen, Bispebjerg and Frederiksberg Hospitals, University of Copenhagen, Bispebjerg Bakke 23, 2400 NV Copenhagen, Denmark; ^3^National Research Centre for the Working Environment, Lersø Parkallé 105, 2100 Copenhagen, Denmark; ^4^Institute of Sports Science and Clinical Biomechanics, SDU Muscle Research Cluster (SMRC), University of Southern Denmark, Campusvej 55, 5230 Odense, Denmark; ^5^Section for Sports Traumatology, Department of Orthopedic Surgery, Bispebjerg and Frederiksberg Hospitals, University of Copenhagen, Bispebjerg Bakke 23, 2400 NV Copenhagen, Denmark

## Abstract

*Objective*. Persistent weakness is a common problem after anterior cruciate ligament- (ACL-) reconstruction. This study investigated the effects of high-intensity (HRT) versus low-intensity (LRT) resistance training on leg extensor power and recovery of knee function after ACL-reconstruction. * Methods*. 31 males and 19 females were randomized to HRT (*n* = 24) or LRT (*n* = 26) from week 8–20 after ACL-reconstruction. Leg extensor power, joint laxity, and self-reported knee function were measured before and 7, 14, and 20 weeks after surgery. Hop tests were assessed before and after 20 weeks. * Results*. Power in the injured leg was 90% (95% CI 86–94%) of the noninjured leg, decreasing to 64% (95% CI 60–69%) 7 weeks after surgery. During the resistance training phase there was a significant group by time interaction for power (*P* = 0.020). Power was regained more with HRT compared to LRT at week 14 (84% versus 73% of noninjured leg, resp.; *P* = 0.027) and at week 20 (98% versus 83% of noninjured leg, resp.; *P* = 0.006) without adverse effects on joint laxity. No other between-group differences were found. * Conclusion*. High-intensity resistance training during rehabilitation after ACL-reconstruction can improve muscle power without adverse effects on joint laxity.

## 1. Introduction

Anterior cruciate ligament (ACL) injuries of the knee are amongst the most common major injuries in sport [1]. People with a high preinjury level of sports participation are often recommended to undergo an ACL-reconstruction [2] and these people are also likely to choose ACL-reconstruction [3]. The goal of a rehabilitation program after an ACL-reconstruction is to regain mobility and muscle function and ultimately to return to sports participation [4]. However, despite postoperative rehabilitation, deficits in muscle function of the operated leg persist up to several years postsurgery [5–12]. These deficits in muscle function are of much concern to clinicians and researchers because a regained muscle function is important for dynamic joint stability [13, 14].

Several studies have demonstrated moderate-to-strong associations (*r* = 0.34–0.74) between thigh muscle strength (primarily quadriceps strength) and knee function (assessed as hop tests) after ACL-reconstruction [15–18]. In addition, it has been shown that inadequate quadriceps strength contributed to altered gait patterns following ACL-reconstruction [19]. People who have regained high levels of quadriceps strength after ACL-reconstruction are more likely to return early to their previous sports activity and at the same level as before the injury [20]. Thus, it seems that quadriceps strength is an important determinant for satisfaction after the ACL-reconstruction [17]. In addition, it has been suggested that quadriceps weakness is a risk factor for developing osteoarthritis [21–23].

There is still no consensus regarding the optimal rehabilitation program after ACL-reconstruction [4, 24]. Current programmes emphasize full passive knee extension, immediate weight bearing as tolerated, and functional exercises [4, 25]. It has been suggested that these programs focus too much on functional low-intensity and sports-specific exercises and that weight training intensity may be too low to increase muscle strength to a satisfactory level [26, 27]. Most studies have measured muscle strength (force) after ACL-reconstruction; however, muscle power (force × velocity) may provide a more sensitive and sports-specific measure of muscle function [5, 11, 28, 29]. The effectiveness of traditional resistance training methods for developing maximal power has been questioned because this type of training has been suggested to increase maximal strength at slow movement velocities rather than improving other components contributing to maximal power production [29].

The aim of the ACL-reconstruction surgery is to create a mechanically stable knee and the aim of the rehabilitation is to create a functionally stable knee. The effects of various rehabilitation exercises in both open and closed kinetic chain have been discussed extensively in the literature, but few prospective randomized studies following ACL-reconstruction have been conducted to investigate these issues [30]. The concern has mainly been about the risk of elongation of the ACL graft and mechanical instability of the knee resulting from early isolated quadriceps resistance training [31]. There is a consensus that open kinetic chain exercises with a focus on endurance do not increase graft laxity and have favourable effects on quadriceps strength, but there is still uncertainty about the optimal timing of introduction of these open kinetic chain exercises [24].

To our best knowledge, the effect of high-intensity and low-intensity resistance training on muscle function in individuals who have undergone ACL-reconstruction has not been investigated previously. Therefore, the objective of the present study was to investigate whether individuals, who perform high-intensity resistance training (HRT) as part of their rehabilitation after ACL-reconstruction, will achieve greater improvements in leg extensor muscle power and greater improvements in knee function compared with individuals performing low-intensity resistance training (LRT) without any negative effect on mechanical instability. We hypothesized that HRT would be superior to LRT for regaining muscle power and knee function, respectively.

### 1.1. Study Design

The study was designed as a single blinded, randomized, clinical trial of two types of resistance training as part of the rehabilitation program after ACL-reconstruction. All participants gave written informed consent before taking part in the study. The study protocol was in compliance with the Helsinki Declaration and the study was approved by the local ethics committee (KF01-008/04).

## 2. Material and Methods

### 2.1. Participants

**Figure 1:**
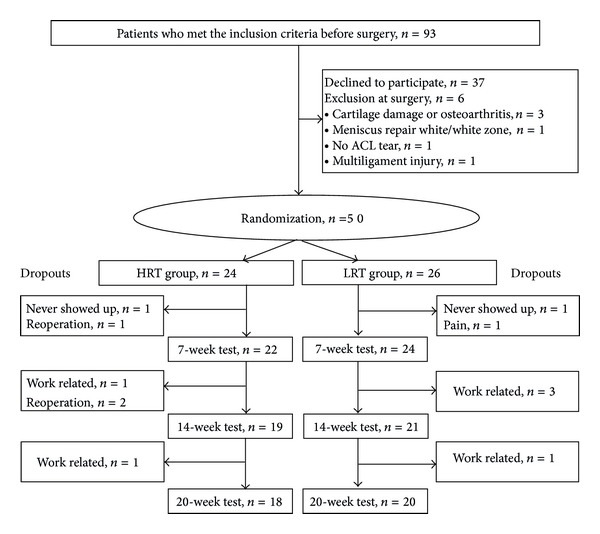
Trial profile. 50 patients were randomized and 6 from each group dropped out of the study. None of the dropouts were related to adverse effects of the strength training. Work related dropouts were because participants were unable to attend group training during office hours.

Men and women aged 18–45 years with isolated ACL rupture who underwent an elective primary ACL-reconstruction and subsequent rehabilitation at Bispebjerg Hospital were recruited consecutively via the operating room list. Subjects were excluded if they had (1) bilateral ACL injury, (2) a previous ACL-reconstruction, (3) repair of meniscus in the index knee within the last 5 months, or (4) earlier intra-articular fracture or osteoarthritis of the knee or (5) if the conventional rehabilitation program could not be followed (Figure 1).

### 2.2. Surgical Procedures

ACL-reconstructions with the bone-patellar tendon-bone or hamstrings tendon (four-leg semitendinosus-gracilis) grafts were carried out by experienced orthopaedic surgeons. The 20-week rehabilitation program was independent of graft choice. Studies comparing ACL-reconstruction using either of these two grafts have shown very similar clinical results [32–34]. However, there is evidence of a greater deficit in knee extensor muscle strength following ACL-reconstruction with patella tendon graft and a lower deficit in knee flexor muscle strength following ACL-reconstruction with hamstrings graft although not all studies have reported muscle strength differences between the two types of surgery [35, 36]. Immediately after the ACL-reconstruction, the participants were randomized to either HRT or LRT, which started at week 8. A similar distribution of sex, graft, and meniscus repair in the two groups was ensured by using the minimisation method with the aid of a computer program [37] for the randomization.

### 2.3. Rehabilitation Protocol

All participants underwent a standardised 20-week rehabilitation program, which started immediately after surgery. The initial focus was on improving postoperative pain and swelling, range of motion, and muscle strength. Full range of motion and weight bearing according to the person's tolerance was allowed and the participants performed isometric quadriceps contractions and dynamic exercises for the hamstring muscles. From week 4 the individuals participated in a supervised one-hour group-based program twice weekly with the main focus on neuromuscular-, functional-, and sports-specific training. Participants who underwent a meniscus repair in combination with the ACL-reconstruction had restricted range of motion during the first five weeks after surgery and were not allowed to start on the group-based program until week 7 but otherwise they received the same program.

A 30-minute progressive, weight training program was initiated 8 weeks after the ACL-reconstruction and was conducted subsequent to the group-based program. The resistance (training loads) was increased when the individual could do more repetitions than the number specified in the weight training protocol [29]. The exercises were performed at a slow speed to ensure full control of the movement. During weight training pain was allowed, but if the participants reported pain of more than 5 on a VAS, range of motion and/or load was reduced. The HRT-program included bilateral and unilateral exercises, that is, leg press (from 90 to 0 degrees in knee), knee flexion in the prone position (0–90 degrees), and seated knee extension (90–0 degrees). The first two weeks of the weight training program served as a familiarization period and thereafter loading increased by lifting weights to failure from 20 to 8 RM (Table 1) with a 2-minute rest period between the sets. However, because the participants had undergone ACL-reconstruction the increase in load happened slowly and high-intensity resistance training started at week 14. The LRT-program included leg press (from 90 to 0 degrees in knee), knee flexion in the prone position (0–90 degrees), and heel raises in the standing position (weight west) with loading increased by lifting loads to failure from 30 to 20 RM (Table 1) and a 1-minute rest period between the sets.

### 2.4. Data Recording

The participants were assessed by the same blinded investigator before and 7, 14, and 20 weeks after surgery. Three external physical therapists, blinded for group allocation, completed all the measurements in the same standardised way regarding test protocol and order of measurements. Before the assessments of muscle power and knee function, the participants completed a 10-minute warmup on a stationary bike. The nonoperated healthy leg was always tested first. Any pain during the tests was measured on a VAS and registered. At the pretest, data regarding preinjury sports were collected.

#### 2.4.1. Objective Outcome Measures

Knee joint laxity was evaluated with the KT-2000 arthrometer (MEDmetric Corporation, San Diego, CA) at 15 Lbs (67N) and 20 Lbs (89N) anterior-posterior directed loads [38]. The measurements continued until the value was reproduced. The value of the 20 Lbs test was used for statistical analyses. KT-2000 instrumented examination of knee laxity in the ACL injured leg has shown relatively high intratester reliability (ICC = 0.95) [38].

Measurements of maximal leg extensor muscle power (force × velocity) were performed using the Leg Extensor Power Rig (Queen's Medical Centre, Nottingham University, UK) according to procedures described elsewhere [39]. In brief, leg muscle power was measured during unilateral leg extension with the participants seated with a flexed knee and the foot positioned on the dynamometer pedal. The free foot rested on the floor. The participants were instructed to push the pedal forward as hard and fast as possible. The extension movement took 0.25–0.4 seconds and the final angular velocity of the flywheel was used to calculate the average leg extensor power produced in the push [39]. Measurements were repeated until maximal power output could not be increased further. At least five repetitions were performed and the highest value was used for data analysis.

Knee function was assessed with a one-legged single hop and triple hop test for distance before and 20 weeks after surgery. The two tests were performed by hopping forward as far as possible and landing on the same leg with the hands on the back [40, 41]. Before each hop test, the participants performed two practice trials. The distance hopped was recorded (cm from toe to heel), and the best of three trials for each leg was used for data analysis.

#### 2.4.2. Self-Reported Outcome Measures

Self-reported knee function and knee associated problems were evaluated by use of the knee injury and osteoarthritis outcome score (KOOS) and the Lysholm score and activity level by the Tegner activity scale before and 7, 14, and 20 weeks after surgery. All scores were used as patient-administered surveys.

KOOS is a self-explained patient-administered instrument to assess the patients' opinion about their knee and associated problems [42]. KOOS consists of 5 subscales: (1) pain, (2) other (knee) symptoms, (3) function in daily living (ADL), (4) function in sport and recreation (sport/rec), and (5) knee-related quality of life (QOL) with a score for each continuous subscale from 0 (extreme symptoms) to 100 (no symptoms).

The Lysholm score, which consists of 8 different items: limp, support, pain, instability, locking of the knee, swelling, stair-climbing, and squatting, and the Tegner activity scale were used to assess function and physical activity [43–46].

### 2.5. Statistical Analyses

Since leg extensor power assessed by the Power Rig device has never been used as a study outcome measure in people with ACL injury or -reconstruction, a prior sample size was calculated to *n* = 20 in each group based on knee extension muscle strength [20] and one-legged single hop for distance with a requirement of a MIREDIF at 20%, power at 80%, and a 5% significance level and allowing for a drop out of 20% in both groups.

Results for objective outcome measures are presented as least square mean, standard error, 95% confidence intervals, and self-reported outcomes measures as median and interquartile range (25–75 percentile). We used *P* < 0.05 as level of significance for testing of main effects and *P* < 0.01 for post hoc tests to account for multiple testing. Between-group differences at baseline were tested with an unpaired *t*-test for objective outcome measures, the Mann-Whitney test for self-reported outcomes measures, and the Chi squared test for numerical data. To determine differences in change over time between groups in the self-reported outcomes the Mann-Whitney test was used. Within-group changes over time were analyzed with Friedman's test or Wilcoxon signed rank test. We used two-way ANOVA (Proc Mixed of SAS version 9.3) to determine differences between groups from before to after surgery and changes during the resistance training phase, respectively. Power for the injured leg was normalized to the baseline value of the noninjured leg (normalized power). Group, time, and group by time were entered in the model as fixed factors. Subject was entered as a random factor. The ANOVA of changes during the resistance training phase was controlled for severity of postsurgery weakness, by including normalized muscle power at week 7 (i.e., first measurement after surgery before initiation of resistance training in both groups) as a covariate. We did not impute missing data as all methods of data imputation have limitations. The mixed procedure of SAS inherently accounts for missing values. Analysis of joint laxity was performed in a similar way, however, not expressed as a percentage of the noninjured leg but as the difference, as this is common for joint laxity.

## 3. Results

Fifty people were randomised (Figure 1) and there were no differences between participants in the HRT- and LRT-groups prior to the ACL-reconstruction except that the time between injury and surgery was longer in the HRT-group (Table 2). Prior to their ACL injury, 76% of the participants had participated in knee-demanding sports such as soccer, team handball, badminton, squash, fencing, martial arts, or basketball. Weekly time spent on sports activities was 4 hours or more for 58% of the participants.

Thirty-eight participants completed the 20-week rehabilitation program (Figure 1). The two graft options were equally represented among the dropouts, that is, 4 ACL-reconstructions with hamstring graft and 2 ACL-reconstructions with patella tendon graft in the HRT- and LRT-groups, respectively. There were no between-group differences in cartilage and meniscus damage but the participants who dropped out had greater preoperative knee laxity in the ACL injured knee (data not shown). This was due to two participants who had very high knee laxity before surgery. At 7 weeks after surgery their knee laxity was similar (i.e., within the same range) to that of the other participants. When the strength training started there was no difference between those who dropped out later (*n* = 10) and those who completed the study (HRT, *n* = 18; LRT, *n* = 20). Otherwise, they did not differ statistically from those who completed the rehabilitation program. Compliance in the two groups was similar; that is, participants in the HRT-group completed on average 22 (19–24) and participants in the LRT-group completed 20 (19–22) out of 24 training sessions.

### 3.1. Maximal Muscle Power

**Figure 2:**
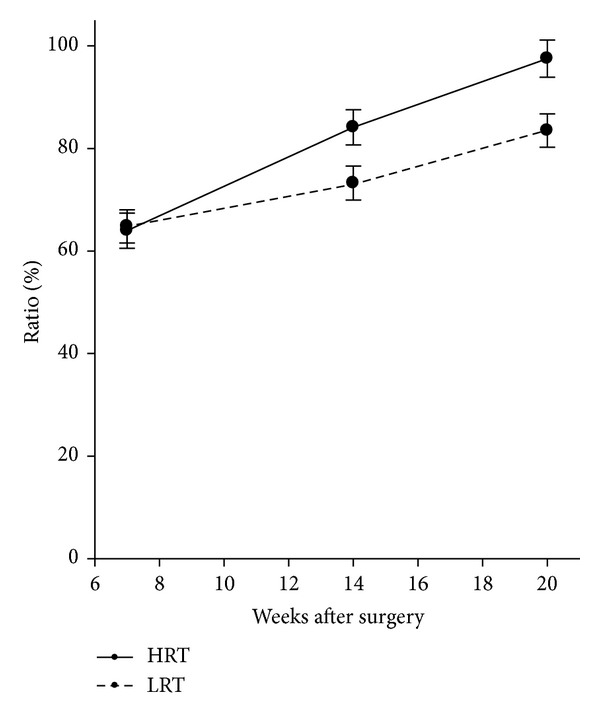
Changes in leg extensor muscle power from pre- to postsurgery. Results in muscle power are presented as least square mean ratios, that is, ACL limb normalized to the presurgery value for the healthy limb multiplied by 100 and SE. There were significant between-group differences at 14 weeks (*P* = 0.027) and at 20 weeks (*P* = 0.006) after surgery.

Before surgery power in the injured leg was 90.1% (CI: 86–96%) of the noninjured leg in both groups. At the first measurements after surgery (i.e., at week 7) this value had decreased to 64.3% (CI: 60–69%) in both groups.  Figure 2 and Table 3 show changes in muscle power from week 7 to 20, that is, during the 12-week resistance training period. During this period we found a significant* group by time* interaction for leg extensor power (*P* = 0.020). Power was regained to a greater extent in HRT-group than LRT-group at weeks 14 and 20, Figure 2 and Table 3.

### 3.2. Knee Joint Laxity

Knee joint laxity was significantly reduced from before to 7 weeks after surgery in both groups and did not change in either of the groups from 7 to 20 weeks. No between-group differences for change in side-to-side difference were found at any time points (Table 3).

### 3.3. Knee Function

The changes in knee function over time did not differ between the groups for one-legged single hop (*P* = 0.566) and triple hop tests (*P* = 0.880). Further, none of the groups had regained their knee function after the intervention period. At twenty weeks after surgery, the ratio was 69.1% ± 5.2 (CI = 58.4; 79.8) and 75.3% ± 4.0 (CI = 67.2; 83.4) for one-legged and triple hop, respectively, in the HRT-group. The ratios were 65.1% ± 5.1 (CI = 54.8; 75.4) and 68.1% ± 3.8 (CI = 60.3; 76.0) for one-legged and triple hop, respectively, in the LRT-group.

### 3.4. Self-Reported Knee Function

During the rehabilitation significant changes occurred for all the subscales in the KOOS (*P* = 0.0001–0.030) and Lysholm score (*P* < 0.0001) but there were no significant differences between the two groups at any time points (Table 4). During the first 7 weeks postoperatively KOOS subscale symptoms decreased significantly (*P* < 0.01) in the LRT-group and function in sport and recreation in the HRT-group. All KOOS subscales except knee-related quality of life increased significantly (*P* < 0.01) in both groups from 7–20 weeks after surgery. The values 20 weeks postoperatively were not significantly different from the presurgery values (*P* = 0.173–0.909), except for the knee-related quality of life, which was higher in the LRT-group (*P* = 0.009). The Lysholm score was unchanged from before surgery to 7 weeks after surgery in both groups, but subsequently increased significantly (*P* < 0.0001) and remained elevated 20 weeks after surgery compared to before surgery (*P* < 0.01) (Table 4).

## 4. Discussion

The present study showed that leg extensor muscle power improved to a greater extent in the HRT-group compared with the LRT-group during the weight training period from 8 to 20 weeks after surgery. Thus, the initial hypothesis that HRT is superior to LRT for regaining muscle power was confirmed by the presented data.

Furthermore, the study demonstrated a substantial decline in leg extensor muscle power in the ACL operated limb 7 weeks after surgery (Figure 2) despite the fact that the rehabilitation program started immediately after surgery. To the best of our knowledge, only one study by Morrissey et al. [47] has documented changes in muscle function during the initial 3 months following ACL-reconstruction. That study demonstrated a knee extensor torque ratio of approximately 0.3 two weeks following ACL-reconstruction with bone-patella tendon-bone graft and approximately 0.5 six weeks after surgery. The most commonly used tool that is reliable in assessing single-joint muscle strength is isokinetic dynamometry [36, 48]. Because seated knee extension in open kinetic chain is the test setup for single-joint muscle strength measurement of quadriceps, the lack of muscle function assessments during the initial 3 months following ACL-reconstruction may be due to concerns about the risk of elongating the ACL graft [31]. In contrast, there may not be the same concern regarding the leg extensor power measurement because that is a multijoint measurement conducted in closed kinetic chain.

The present results indicate that high-intensity resistance training appears to be safe. One concern could be that open kinetic chain, high-intensity knee extensor resistance training would cause anterior knee pain [47], but we had no reports of increased anterior knee pain in the HRT-group. Similarly, Morrissey et al. [47] detected no difference in anterior knee pain between knee extensor and leg extensor resistance training (3 × 20 RM) in the early period (2–6 weeks after surgery) after ACL-reconstruction with bone-patella tendon-bone graft. The fact that HRT resulted in a greater improvement in leg extensor muscle power compared with LRT in our study suggests that this type of training can be recommended in future ACL rehabilitation programs.

Most likely, the superior gains following HRT versus LRT appeared as the result of more marked adaptations in neuromuscular function [28, 49] and/or a greater muscle hypertrophy response [50, 51]. In support of this notion, it has been demonstrated that heavy resistance exercise is highly effective of eliciting enhanced neuromuscular activity [28, 52] and skeletal muscle growth [50, 51]. These effects are less pronounced following resistance training using low external loads [53]. Both groups performed leg press and hamstrings exercises, which only differed in training intensity. In contrast, the HRT-group performed seated knee extension exercise and the LRT-group performed heel raises in the standing position. Because extensor muscles of the knee, hip, and ankle all contribute to the results in leg extensor power [39] it cannot be excluded that both the seated knee extension exercise and the heel raises in the standing position may have influenced the results. On the other hand, the test movements (Nottingham Power Rig procedures) were not employed during training in any of the two intervention groups, resulting in only minimal learning effects.

According to the recommendations from American College of Sports Medicine, two general loading strategies should be used for improving power: (1) strength training and (2) use of low intensity (0–60% of 1 RM) performed at a fast contraction velocity [28, 29]. For safety reasons our participants performed the contractions at a low speed because the weight training started fairly soon after surgery. In healthy adults, low-intensity resistance training with slow movement has not convincingly been shown to improve power or function [28]. It could thus be speculated that the faster improvement in power in the HRT-group may have been due to the heavier loads lifted [49]. This finding is supported by a study on endurance runners [49] which showed that 8 weeks of heavy strength training was more beneficial in improving neuromuscular characteristics than muscle endurance exercise and in particular contributed to improvements in high-intensity running performance. Yet, we had expected a greater between-group difference in muscle power following the training period but the effective period with HRT (load 8 RM) lasted only 6 weeks and this period was probably too short to elicit very marked differences in outcome. On the other hand, a recent study [54] found equal improvements in knee extensor maximal power output, rate of force development, and hypertrophy after 10 weeks of unilateral knee extension resistance training with low intensity (30% of 1 RM) lifted to failure versus high intensity (80% of 1 RM) lifted to failure. Finally, in the late phase of the 20-week rehabilitation program all participants performed plyometric training as part of the group-based program, and this type of training may have had a positive effect on leg extension muscle power [55].

Our second hypothesis was not confirmed since HRT did not appear to regain knee function as evaluated by the hop tests faster. It could be argued that the period with high-intensity resistance exercise was not long enough (load 8 RM, 14 to 20 weeks after surgery) and that a longer training period would have been needed to ensure transferability from increased muscle function to improvement in the hop tests. Further, the plyometric training, which was performed in both groups, may have contributed to this lack of between-group differences [55]. Moreover, the hop test is more complex than the Leg Extensor Power Rig test as it requires power, balance, and coordination. Finally, fear avoidance may have played a role [56]. The relationship between single-leg hop capabilities, muscle function, and anterior knee joint laxity in conjunction with fear of movement and reinjury has not been investigated in patients following ACL-reconstruction [56]. According to the postoperative regimen at our hospital participation in knee-demanding sports was not allowed until 9 to 12 months after surgery. Therefore, fear of painful reinjury may have caused the patients to avoid behaviours that would potentially increase the risk of reinjury.

The mechanical stability of the knee increased as a result of the ACL-reconstruction, which is consistent with results of previous studies [57, 58]. Importantly, our results suggest that neither low-intensity nor high-intensity resistance training has an adverse effect on knee joint stability since there were no significant changes in knee laxity during the weight training period.

Regarding self-reported outcomes there were no significant differences between the groups. Both groups increased in Lysholm score from before surgery to 20 weeks after surgery (Table 4). The presurgery values correspond to those documented in patients with abnormal or severely abnormal overall knee function while the values 20 weeks after surgery correspond to normal or nearly normal overall knee function [43].

In contrast, results in all KOOS subscales were the same before surgery and 20 weeks after surgery (Table 4). Roos et al. [42] showed a large effect size for the KOOS instrument 6 months after surgery and the largest effect size for the subscales “function in sport and recreation” and “knee-related quality of life” [42]. However, because our participants were not allowed to participate in knee-demanding sports 6 months after surgery restrictions in sports may have resulted in lower scores in the subscales “function in sports and recreation” and “knee-related quality of life.”

### 4.1. Strength and Limitations

The strength of our study is that we were able to document the substantial decline in muscle power during the first 7 weeks after surgery and the subsequent recovery in muscle power of the ACL-reconstructed limb by means of the Leg Extensor Power Rig. This measuring equipment has not previously been used to test muscle function after ACL injury or ACL-reconstruction. However, the Nottingham Power Rig has been used to evaluate muscle function in healthy people, aged 20–86 [39, 59], and in a wide range of individuals with known musculoskeletal pathologies/deficits, including geriatric patients after proximal femoral fracture [60]. The assessment is not time consuming; it is easy and safe to perform for all age groups and levels of physical capacity [39], while at the same time being reproducible (CV = 9–13%) in healthy people, aged 20–86 [39]. The present results demonstrate that this method can discriminate between muscle power in the ACL limb and the healthy limb up to 20 weeks after surgery as well as detect training induced changes in this parameter over time. This implies that the Leg Extensor Power Rig may be used in the early phase of the rehabilitation program to assess the status and progress the rehabilitation after ACL tear or -reconstruction. However, the reliability and agreement as well as the validity of the leg extensor power measurement need to be determined in people with ACL injuries.

Our study has some limitations. First, we had a high number of dropouts, which means that we did not reach the desired number of patients in each group. This weakens the statistical power of our results. On the other hand, a small sample size would probably make it more difficult to document a longitudinal effect of training. The participants who dropped out had greater preoperative knee laxity in the ACL injured knee compared to those who completed the rehabilitation program. Since there were no differences between the groups when the strength training started, we do not think that the difference in laxity before surgery is of significant importance. Further, the HRT-group had significantly longer time between injury and surgery than the LRT-group. This could have resulted in more degenerative changes, that is, meniscus and cartilage damage in the HRT-group, but no between-group differences in cartilage and meniscus damage were reported. In addition, the weight training in the two groups did not consist of exactly the same exercises and was not entirely matched for total training volume. The limitations mentioned imply that our results may not be generalizable to all patients going through an ACL-reconstruction.

## 5. Conclusion

The present data indicate that high-intensity resistance training as part of early rehabilitation after ACL-reconstruction may contribute to a faster recovery of leg extension muscle power compared with low-intensity resistance training without introducing any adverse effect on knee joint stability. Most likely, the accelerated/amplified gains observed with high-intensity resistance training were caused by more marked neuromuscular adaptations and/or greater muscular regrowth induced by this training modality.

## Figures and Tables

**Table 1:** The 12-week weight training protocol for the high- and low-intensity resistance training.

Week	HRT-group Sets × repetitions (load)		LRT-group Sets × repetitions (load)	
	Bilateral	Unilateral	Bilateral	Unilateral
8*	1 × 20 (20RM)	2 × 15 (20RM)	1 × 30 (30RM)	1 × 20 (30RM)
9*	1 × 20 (20RM)	3 × 15 (20RM)	1 × 30 (30RM)	2 × 20 (30RM)
10 + 11	1 × 15 (15RM)	3 × 12 (15RM)	1 × 20 (20RM)	2 × 20 (20RM)
12 + 13	1 × 12 (12RM)	3 × 10 (12RM)	1 × 20 (20RM)	2 × 20 (20RM)
14–20	1 × 8 (8RM)	3 × 8 (8RM)	1 × 20 (20RM)	2 × 20 (20RM)

HRT: high-intensity resistance training; LRT: low-intensity resistance training.

RM**:** repetition maximum. 1RM is the most weight you can lift for one repetition. 15RM is the most weight you can lift for 15 repetitions.

*Familiarization period.

**Table 2:** Baseline characteristics of the participants 1-2 weeks before ACL-reconstruction.

Variable	HRT-group	LRT-group	*P* value
Number (*n*)	24	26	
Age (year)	29.2 ± 1.5	29.2 ± 1.1	0.976
Sex: M/F (*n*)	15/9	16/10	0.994
Body weight (kg)	76.1 ± 2.7	77.2 ± 2.6	0.754
Graft: BPTB/STG	13/11	14/12	0.982
Months from injury to surgery	40.3 ± 10.0	16.8 ± 4.9	0.043
Meniscus tear (*n*)	11	13	0.877
Repair with arrows	5	6	
Resection, current/previous	6	7	
Cartilage damage (*n*)	7	10	0.448
Leg extensor muscle power			
Ratio ACL/healthy limb %	90.5 ± 2.8	89.4 ± 3.9	0.814
Knee joint laxity			
Diff. ACL and healthy limb (mm)	2.1 ± 0.3	2.8 ± 0.4	0.184
One-legged single hop			
Ratio ACL/healthy limb %	79.7 ± 4.7	68.8 ± 3.7	0.077
One-legged triple hop			
Ratio ACL/healthy limb %	86.4 ± 3.5	82.1 ± 3.2	0.367
Tegner activity scale (0–10)	3 (2–5)	2 (2–4)	0.292
Lysholm score (0–100)	70 (52–83)	66 (56–81)	0.771
KOOS (0–100)			
Pain	85 (67–94)	79 (67–90)	0.514
Symptoms	89 (70–96)	80 (62–90)	0.131
ADL	93 (75–97)	89 (79–97)	0.936
Sport	70 (49–76)	60 (40–81)	0.499
QOL	44 (38–56)	44 (36–50)	0.467

Data are reported as mean ± SE except self-reported surveys, which are presented as median (interquartile range).

HRT: high-intensity resistance training; LRT: low-intensity resistance training; BPTB: bone-patellar tendon-bone graft; STG: four-legged semitendinosus-gracilis graft; KOOS: knee injury and osteoarthritis outcome score with subscales (0: extreme symptoms, 100: no symptoms): pain, other symptoms, ADL: function in daily living, sport/rec: function in sport and recreation, and QOL: knee-related quality of life.

**Table 3:** Change in muscle power and knee joint laxity during the intervention period.

	7 weeks after surgery		14 weeks after surgery		20 weeks after surgery	
	HRT	LRT	HRT	LRT	HRT	LRT
Leg extensor power	64.0 ± 3.4 (57.1; 70.8)	64.8 ± 3.2 (58.3; 71.3)	84.1 ± 3.4* (77.3; 91.0)	73.3 ± 3.3 (66.6; 79.9)	97.5 ± 3.6** (90.3; 104.7)	83.5 ± 3.2 (77.0; 90.0)
Knee joint laxity	1.2 ± 0.3 (0.7; 1.7 )	1.3 ± 0.2 (0.8; 1.8 )	1.3 ± 0.3 (0.8; 1.8)	0.9 ± 0.3 (0.4; 1.4)	1.4 ± 0.3 (0.9; 2.0)	1.1 ± 0.3 (0.6; 1.6)

Power results are presented as least square mean ratios (ACL limb normalized to the presurgery value for the healthy limb multiplied by 100) ± SE (CI). Knee joint laxity results are presented as least square mean side-difference in mm ± SE (CI).

HRT: high-intensity resistance training; LRT: low-intensity resistance training. Significant between-group differences in regain of muscle power: *P = 0.027, **P = 0.006.

**Table 4:** Results self-reported data before surgery and 7 and 20 weeks after the ACL-reconstruction.

	Presurgery		7 weeks after surgery		20 weeks after surgery	
	HRT	LRT	HRT	LRT	HRT	LRT
Lysholm score	70 (47–81)	63 (57–80)	60 (49–67)	62 (61–74)	80 (66–84)	80 (74–85)
Tegner score	3 (2–5)	2 (2–4)	2 (2-2)	2 (2-2)	4 (2–5)	3 (2–4)
KOOS						
Pain	80 (64–94)	81 (72–92)	69 (63–75)	75 (64–78)	83 (65–93)	81 (78–89)
Symptoms	89 (68–96)	75 (64–89)	64 (57–80)	54 (46–64)	86 (68–96)	79 (68–89)
ADL	93 (78–96)	89 (85–97)	78 (73–88)	85 (72–91)	91 (83–98)	93 (90–96)
Sport/rec	70 (43–75)	70 (40–85)	35 (20–40)	35 (25–55)	60 (43–73)	55 (45–70)
QOL	44 (38–56)	50 (38–50)	44 (28–50)	44 (38–56)	50 (34–66)	50 (44–63)

Results are presented as median and interquartile range.

HRT: high-intensity resistance training; LRT: low-intensity resistance training; KOOS: knee injury and osteoarthritis outcome score with subscales (0: extreme symptoms, 100: no symptoms): pain, other symptoms, ADL: function in daily living, sport/rec: function in sport and recreation, and QOL: knee-related quality of life.
